# Myocardial Ischemic Postconditioning Promotes Autophagy against Ischemia Reperfusion Injury via the Activation of the nNOS/AMPK/mTOR Pathway

**DOI:** 10.3390/ijms18030614

**Published:** 2017-03-11

**Authors:** Maojuan Hao, Suhua Zhu, Liang Hu, Hongyi Zhu, Xiaowei Wu, Qingping Li

**Affiliations:** Department of Pharmacology, Jiangsu Provincial Key Lab of Cardiovascular Diseases and Molecular Intervention, Nanjing Medical University, Nanjing 211166, China; maojhao@163.com (M.H.); zshnjmu@163.com (S.Z.); zhuhongyi0217@sina.cn (H.Z.); 15240242832@sina.cn (X.W.)

**Keywords:** ischemic postconditioning (IPostC), ischemia/reperfusion, autophagy, neuronal nitric oxide synthase (nNOS), adenosine monophosphate-activated protein kinase (AMPK), mammalian target of rapamycin (mTOR)

## Abstract

Autophagy participates in the progression of many diseases, comprising ischemia/ reperfusion (I/R). It is reported that it is involved in the protective mechanism of ischemic postconditioning (IPostC). According to research, neuronal nitric oxide synthase (nNOS) is also involved in the condition of I/R and IPostC. However, the relationship between nNOS, autophagy and IPostC has not been previously investigated. We hypothesize that IPostC promotes autophagy activity against I/R injury partially through nNOS-mediated pathways. Mouse hearts were subjected to I/R injury through the ligation of the left anterior descending coronary artery. H9c2 cells were subjected to hypoxia/reoxygenation (H/R) in vitro. IPostC, compared with I/R, restored nNOS activity, increased the formation of autophagosome and restored the impaired autophagic flux, thus autophagic activity was raised markedly. IPostC increased adenosine monophosphate-activated protein kinase (AMPK) phosphorylation and suppressed mammalian target of rapamycin (mTOR), but a selective nNOS inhibitor abolished those effects. Similar effects of IPostC were demonstrated in H9c2 cells in vitro. IPostC decreased infarct size and preserved most of the normal structure. The level of reactive oxygen species (ROS) and cell apoptosis were reduced by IPostC with improved cell viability and mitochondrial membrane potential. However, an autophagy inhibitor suppressed the protective effects. These results suggest that IPostC promoted autophagy against I/R injury at least partially via the activation of nNOS/AMPK/mTOR pathway.

## 1. Introduction

Ischemic heart disease has a high morbidity and mortality; it is the leading cause of death throughout the world [1]. Rapid reperfusion of the ischemic myocardium is still the current standard treatment for myocardial infarction [2]. However, it potentially causes additional injury in the early period, such as disturbances in ionic homeostasis, reactive oxygen species (ROS) over production, inflammatory responses, mitochondrial dysfunction and calcium overload. These are collectively referred to as myocardial ischemia/reperfusion (I/R) injury [3]. Currently, there is still no clinically effective way to reduce I/R injury. Extensive research has focused on increasing heart tolerance to I/R injury using conditioning strategies such as ischemic preconditioning (IPre) [4]. A large number of studies have confirmed that IPre has a protective effect on I/R hearts. However, due to the unpredictable nature of ischemic heart disease, and the fact that implementation of IPre needs to be carried out before ischemia, the clinical application of IPre is greatly limited. More reliable treatments are urgently needed to relieve I/R injury.

Recent studies have shown that ischemic postconditioning (IPostC) significantly protects cardiomyocytes against I/R injury [5]. However, its protective mechanism is not fully understood. Considering the clinical operability and great clinical significance [6], it is necessary to further clarify the mechanism of IPostC in alleviating I/R injury.

Studies have indicated that autophagy plays a part in myocardial I/R. Autophagy is the process of phagocytosing and degrading autologous cytoplasmic proteins or organelles, thereby accomplishing the metabolic needs of the cells themselves and the renewal of some organelles [7]. It is a key regulator of I/R injury and is believed to play important roles in the heart during I/R [8]. Some current research show that autophagy plays a dual role in myocardial I/R injury. Autophagy is activated in response to energy crisis. Enhancing autophagic activity within moderate range may compensate for mitochondrial injury and contribute to proteostasis in I/R injury [9,10]. Some studies found that autophagy clearance was dramatically decreased with reperfusion in cardiomyocytes, which is detrimental to cardiomyocyte survival during reperfusion [11]. Therefore, restoration of impaired autophagic flux maybe an effective way to alleviate I/R injury.

Autophagy is also reported to be regulated by IPostC and may be involved in the protective mechanism of IPostC [12,13]. IPostC exerts cardioprotective effects partially by alleviating oxidative stress; autophagy promoted by ROS during reperfusion may also reduce the level of ROS. Therefore, autophagy may participate in the cardioprotection of IPostC through alleviating oxidative stress.

Some research demonstrated that neuronal nitric oxide synthase (nNOS) was cardioprotective in different disease states [14]. Keeping nitroso-redox and Ca^2+^ cycling in balance is important for nNOS to regulate mitochondrial and sarcoplasmic reticulum (SR) function [15,16]. According to our previous research, nNOS is not only involved in the myocardial I/R injury but also participates in the cardioprotection of IPostC by alleviating oxidative stress and calcium overload [17]. Besides, nitric oxide (NO) and overexpression of NOS isoforms have been found to impair autophagy at an early stage of autophagosome synthesis in neurons and cancer cells [18]. Recent studies have also shown a relationship between IPostC and autophagy through the interaction of P70S6 kinase [19], which is the effector of the mammalian target rapamycin (mTOR), while mTOR is negatively regulated by adenosine monophosphate-activated protein kinase (AMPK). NO generated via nNOS in the mitochondria may influence the AMP/ATP ratio and AMPK activity within a cell [20], therefore nNOS may be involved in the regulation of autophagy in IPostC.

However, the relationship of nNOS, autophagy and IPostC has not been previously investigated. We hypothesize that IPostC promotes autophagic activity against reperfusion injury during early reperfusion, partially via the activation of nNOS related pathways.

## 2. Results

### 2.1. IPostC Restored nNOS Activity in Myocadium and H9c2 Cells

**Figure 1:**
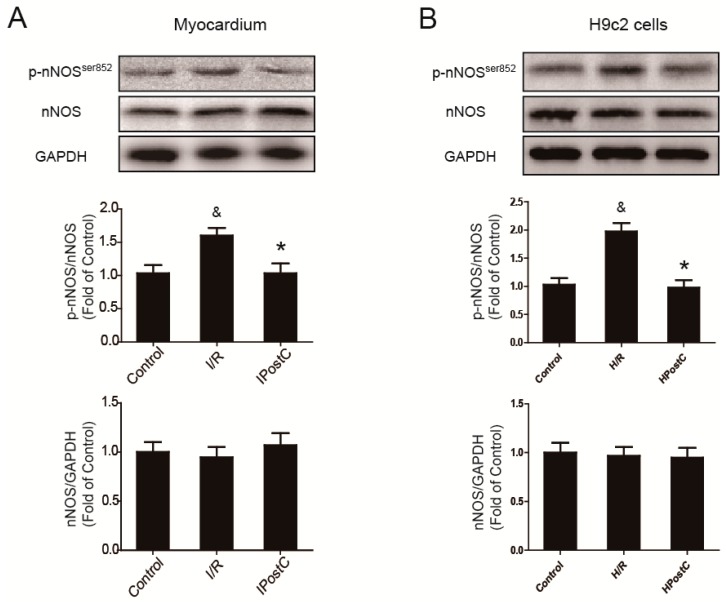
Activity of neuronal nitric oxide synthase (nNOS) was restored by ischemic postconditioning (IPostC) in the myocardium and H9c2 cells. (**A**) Expression of nNOS and nNOS^Ser852^ in the myocardium at 30 min of reperfusion; (**B**) The level of nNOS and nNOS^Ser852^ in H9c2 cells at 30 min of reoxygenation. ^&^ *p* < 0.05 versus control, * *p* < 0.05 versus ischemia/reperfusion (I/R). The mean values ± SEM, *n* = 6.

In order to study whether nNOS is affected by IPostC, the expression of nNOS was measured in myocardium. Compared with the control group, there was no significant change in the expression of total nNOS in the I/R group and IPostC group. However, the I/R group showed an increased level of p-nNOS^Ser852^ where it was reduced in the IPostC group. Similar results were also observed in H9c2 cells (Figure 1). Ser852 is an inactive site of nNOS, therefore the activity of nNOS was partially inhibited at early reperfusion while it was restored by IPostC.

### 2.2. IPostC Promoted Autophagy via nNOS in I/R Injured Myocardium

**Figure 2:**
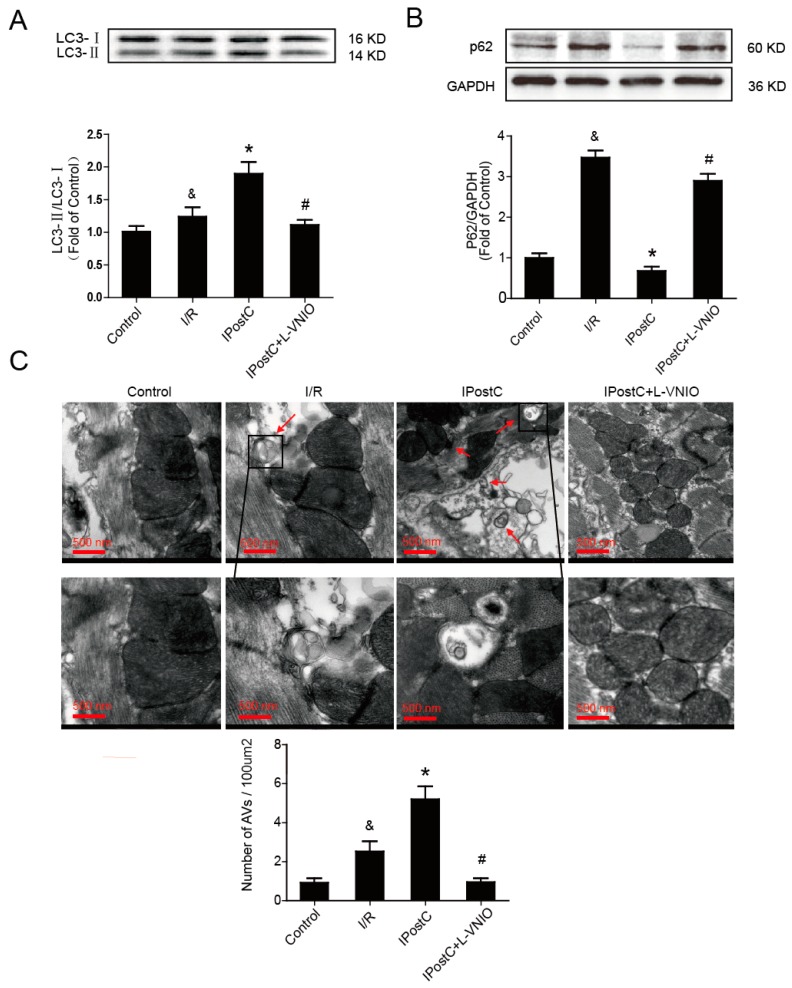
Autophagy activity was promoted by IPostC via nNOS in myocardium. (**A**) The protein expression of LC3 in the myocardium at 30 min of reperfusion; (**B**) The level of p62 was detected in the myocardium; (**C**) Autophagosomes (AVs) which were marked with red arrows were confirmed using electron microscopy in the control, I/R, IPostC group and IPostC treated with nNOS inhibitor L-VNIO. ^&^ *p* < 0.05 versus control, * *p* < 0.05 versus I/R, ^#^ *p* < 0.05 versus IPostC. The mean values ± SEM, *n* = 6.

Autophagy activation is involved in myocardial ischemia. In order to study the role of IPostC in regulating autophagy, we detected the regulation of IPostC to the LC3 protein and p62 in myocardium by Western blot analysis, as these are often used as the indicators of autophagy. The ratio of LC3-II/LC3-I was significantly higher and the level of p62 was lower in the IPostC group than the I/R group. However, the effect of IPostC was suppressed by nNOS inhibitor L-VNIO (Figure 2A,B). Electron microscopy was also used to observe the induction of autophagy. There were almost no autophagosomes and autolysosomes in the control group, however, their levels were increased in the I/R group and were further upregulated by IPostC, which were reduced by the nNOS inhibitor (Figure 2C). In addition, the use of inhibitor L-VNIO alone without IPostC or HPostC had no effect on autophagic activity (Supplementary Figures S1 and S2). These data suggest that IPostC promoted autophagy activity through nNOS-mediated pathways. 

### 2.3. IPostC Enhanced Autophagic Activity via nNOS in H9c2 Cells In Vitro

**Figure 3:**
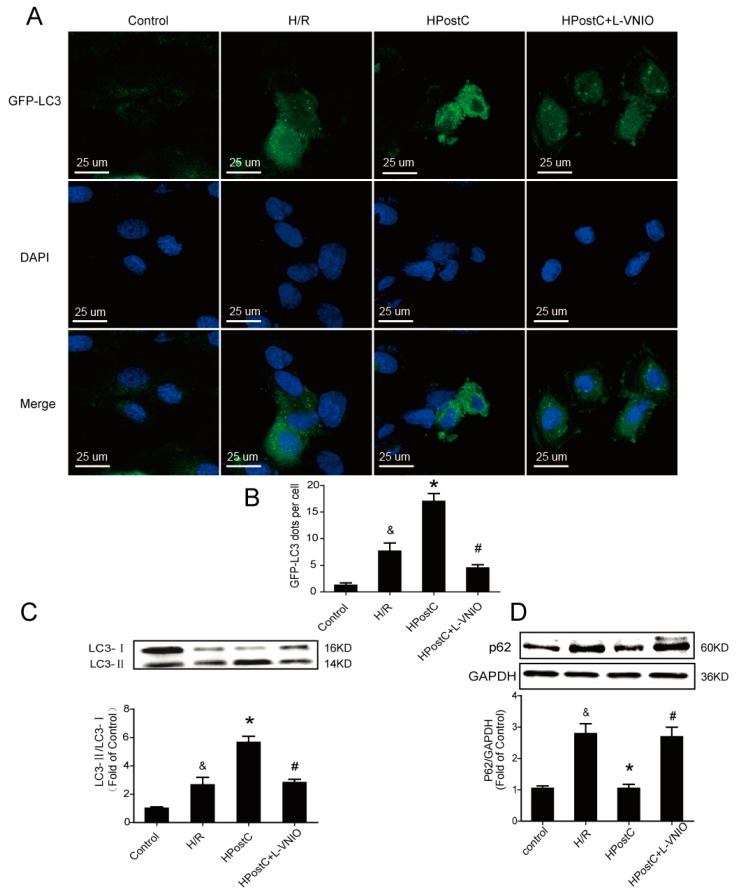
Hypoxic postconditioning (HPostC) enhanced autophagic activity via nNOS in H9c2 cells; (**A**) H9c2 cells were infected with GFP-LC3 adenovirus. The nuclei were labeled with DAPI (blue); (**B**) The statistics of GFP-LC3 dots; (**C**) Expression of LC3 in H9c2 cells at 30 min of reoxygenation; (**D**) Expression of p62 at 30 min of reoxygenation. ^&^ *p* < 0.05 versus control, * *p* < 0.05 versus hypoxia/reoxygenation (H/R), ^#^ *p* < 0.05 versus HPostC. The mean values ± SEM, *n* = 6.

In order to further certify our surmise that IPostC promotes autophagy clearance and enhances autophagy activity, adenovirus encoding GFP-LC3 was used to infect H9c2 cells. When autophagy is induced, the GFP-LC3 fusion protein is translocated to the autophagic membrane and forms a number of bright green fluorescent spots under the fluorescence microscope. As shown in Figure 3A,B, there were almost no GFP-LC3 dots in the control group. Hypoxic postconditioning (HPostC) significantly increased the number of GFP-LC3 dots compared with the hypoxia/reoxygenation (H/R) group. However, nNOS inhibitior abolished the effect of HPostC. To further investigate the effect of HPostC and nNOS on autophagy, expression of LC3 and p62 in H9c2 cells were also detected by Western blot analysis. The ratio of LC3-II/LC3-I was significantly upregulated and the p62 protein level was decreased in the HPostC group compared with the H/R group, whereas administration of the nNOS inhibitor L-VNIO down-regulated the expression of LC3-II and increased the expression of p62 (Figure 3C,D). Similar changes were observed in cells that used nNOS-siRNA (Supplementary Figure S3). Collectively, these data indicate that autophagic activity and autophagy clearance promoted by IPostC via nNOS were involved both in vitro and in an animal model.

### 2.4. Activation of the nNOS/AMPK/mTOR Signaling Pathway Was Involved in the Regulation of IPostC on Autophagy

**Figure 4:**
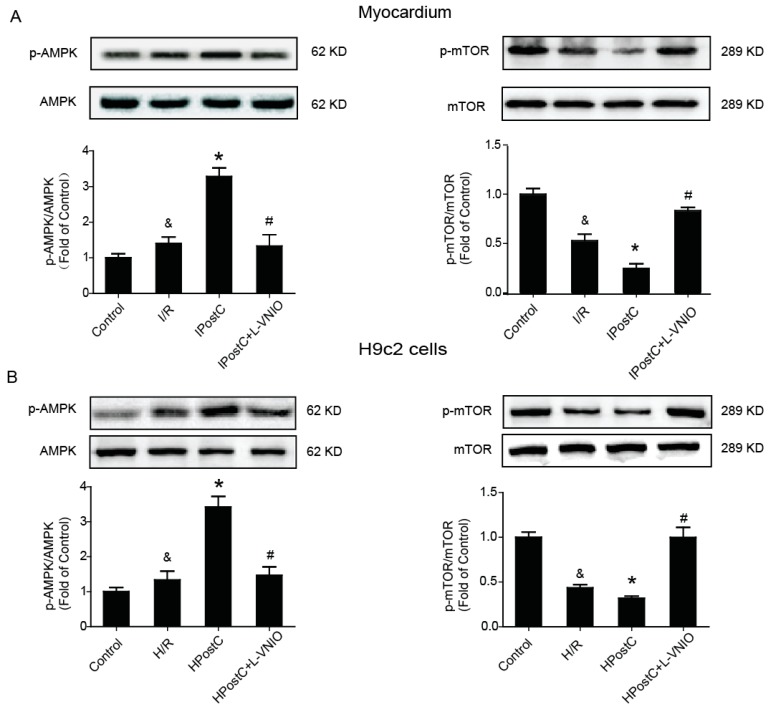
IPostC activated the nNOS/AMPK/mTOR signaling pathway during early reperfusion. (**A**) The protein expression and optical density analysis of p-AMPK (Thr172)/AMPK, p-mTOR(Ser2448)/mTOR in the myocardium at 30 min of reperfusion; (**B**) Expression of p-AMPK, p-mTOR in H9c2 cells at 30 min of reoxygenation. ^&^ *p* < 0.05 versus control, * *p* < 0.05 versus I/R, ^#^ *p* < 0.05 versus IPostC. The mean values ± SEM, *n* = 6.

The kinase mTOR, which is a key regulator of autophagy induction [21], plays an important role in the progress of autophagy. When mTOR is inhibited (AMPK and p53 signaling), autophagy is induced. AMPK is a key regulator of bioenergetic metabolism [22]. In the state of hypoxia and ischemia, the ratio of AMP/ATP increases and it stimulates α-Thr172 phosphorylation, then AMPK is activated and mTOR is suppressed. AMPK/mTOR signaling is involved in the regulation of autophagy during ischemia, but it is unclear whether the signaling is also involved in the regulation of IPostC on autophagy. To explore the possibility that IPostC promotes autophagy activity via nNOS–mediated pathway, we measured the expression of p-AMPK (Thr172) and p-mTOR (Figure 4A). I/R increased AMPK phosphorylation and reduced mTOR phosphorylation in the myocardium. IPostC further enhanced the level of p-AMPK and depressed the expression of p-mTOR while nNOS inhibitor abolished the effect of IPostC. The levels of p-AMPK/AMPK and p-mTOR/mTOR were then confirmed in H9c2 cells, and consistent with our in vivo study (Figure 4B and Supplementary Figure S3D). Taken together, these findings indicate that the regulation of IPostC on autophagy was partially through the nNOS/AMPK/mTOR signaling pathway.

### 2.5. Autophagy Inhibitor Abolished the Protective Effect of IPostC in the Myocardium

**Figure 5:**
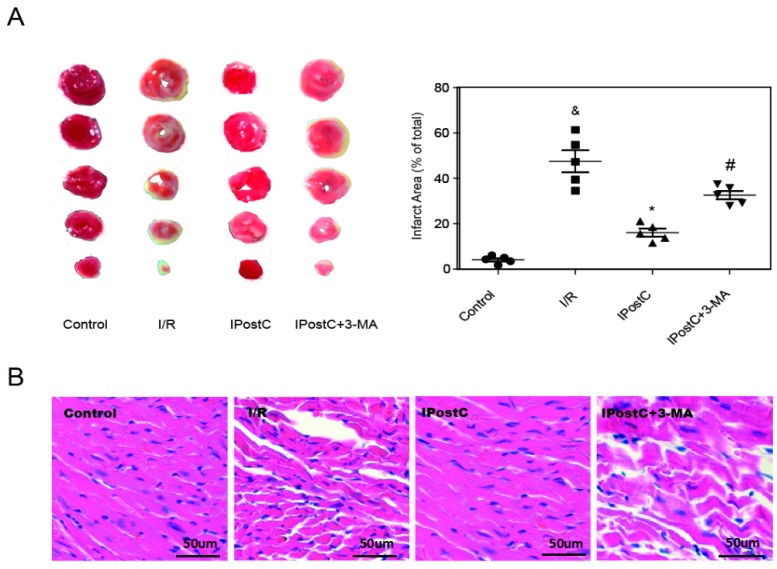
The protective effect of IPostC in the myocardium was inhibited by an autophagy inhibitor. (**A**) Infarct size was measured at 120 min of reperfusion; (**B**) Representative histological images of hearts at 120 min of reperfusion. ^&^ *p* < 0.05 versus control, * *p* < 0.05 versus I/R, ^#^ *p* < 0.05 versus IPostC. The mean values ± SEM, *n* = 6.

In order to detect whether autophagy is important for the protective effect of IPostC, a classical autophagy inhibitor 3-MA, which is able to inhibit the formation of autophagosomes, was applied to the study. Mice were pretreated with 3-MA 30 min prior to coronary artery occlusion. Following 2 h reperfusion, the infarct size in the hearts subjected to I/R was 43.6% ± 2.5%, and this was reduced to 16.8% ± 2.1% (*p* < 0.05) in hearts subjected to IPostC. By contrast, the infarct size in the IPostC + 3-MA mice was significantly increased to 37.2% ± 1.8% (*p* < 0.05 versus IPostC group; Figure 5A). In addition, representative histological images of hearts were taken after 2 h of reperfusion. Obvious changes such as a grossly distorted structure, interstitial edema, and frequent contraction band appearance were observed in the I/R group, whereas normal structures were largely preserved in the IPostC group. The cardioprotection of IPostC was abolished by the autophagy inhibitor 3-MA, as cardiomyocyte necrosis, nuclear dissolution and severely deformed structures were discovered in the IPostC + 3MA group (Figure 5B). The use of inhibitor 3-MA alone without IPostC or HPostC had no effect on myocardium and H9c2 cells (Supplementary Figure S4). These results indicate that the inhibition of autophagy abolished the cardioprotection of IPostC.

### 2.6. IPostC Protected H9c2 Cells by Promoting Autophagy In Vitro

**Figure 6:**
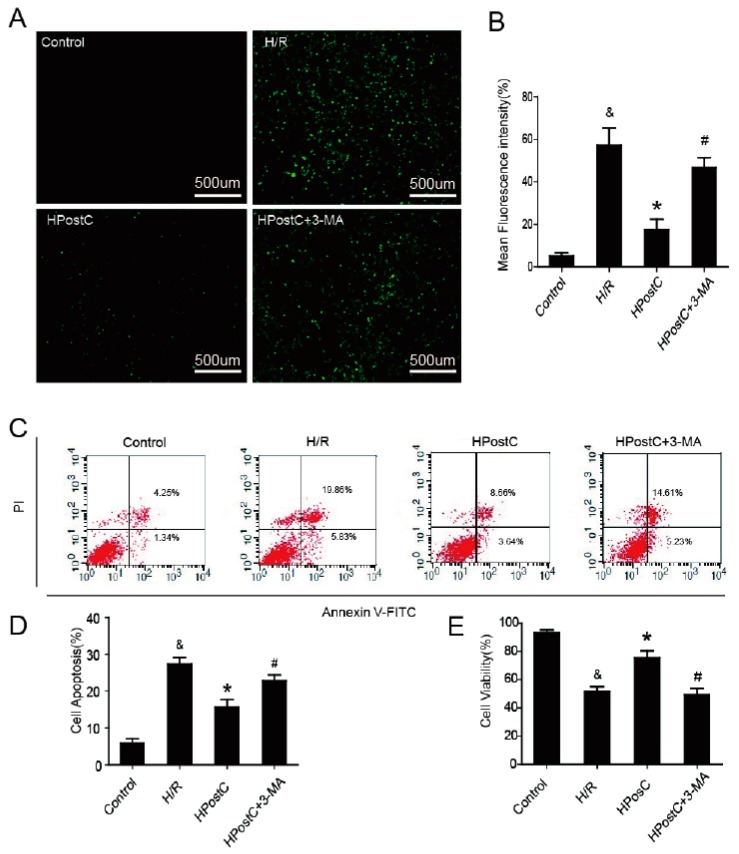
HPostC protected H9c2 cells by promoting autophagy in vitro. (**A**,**B**) ROS production was detected in H9c2 cells at 30 min of reoxygenation; (**C**) H9c2 cells were collected and stained with annexin V-FITC/propidium iodide and detected by flow cytometry; (**D**) A bar diagram of the cell apoptosis; (**E**) Cell viability was assessed by CCK-8 kits. ^&^ *p* < 0.05 versus control, * *p* < 0.05 versus H/R, ^#^ *p* < 0.05 versus HPostC. The mean values ± SEM, *n* = 6.

Oxidative stress is a major factor of I/R injury. To explore whether autophagy plays a role in the protection of IPostC against oxidative stress, ROS production was measured (Figure 6A,B). Compared with the H/R group, HPostC significantly reduced the generation of ROS in H9c2 cells. However, autophagy inhibitor 3-MA abolished the protection of HPostC. OONO^−^ is a RNS that is also a major cytotoxic factor involved in myocardial I/R injury. Nitrotyrosine, which is a footprint of OONO^−^ formation was measured at 30 min of reoxygenation. We found that there was little production of nitrotyrosine in these groups and the data indicate that RNS was not mainly responsible for H/R injury during early reperfusion (Supplementary Figure S5). To further confirm our hypothesis that the cardioprotection of IPostC is related to the activation of autophagy, cell apoptosis and viability were measured in H9c2 cells by flow cytometry and CCK-8 test kits (Figure 6C–E). Compared with the H/R group, HPostC reduced apoptosis and increased cell activity, while 3-MA inhibited the effects. These data suggest that HPostC exerted its beneficial effect partially via the activation of autophagy.

### 2.7. IPostC Improved Mitochondrial Function by Enhancing Autophagic Activity

**Figure 7:**
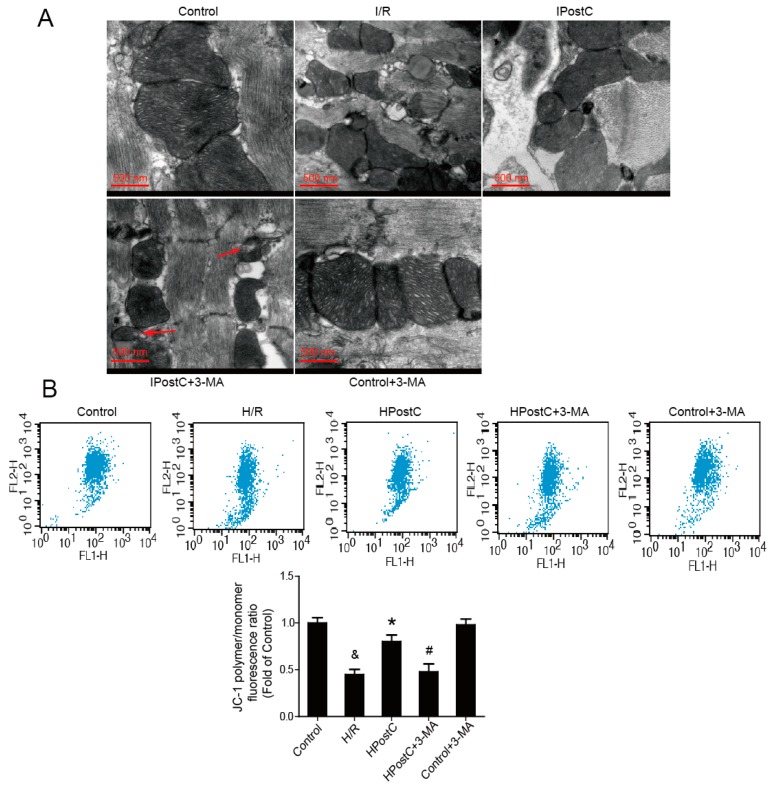
Determination of mitochondrial function. (**A**) Mitochondrial morphology was observed under electron microscopy in the myocardium (mitochondrial vacuoles were indicated by red arrows); (**B**) Measurement of mitochondria membrane potential after 30 min of reoxygenation. ^&^ *p* < 0.05 versus control, * *p* < 0.05 versus H/R, ^#^ *p* < 0.05 versus HPostC. The mean values ± SEM, *n* = 6.

The injured mitochondria would further contribute to myocardial I/R injury. In order to investigate whether IPostC and autophagy can improve mitochondrial function, mitochondrial morphology was observed under electron microscopy. Smaller and fragmented mitochondria were observed in the I/R group while most of the normal mitochondria were preserved after IPostC. 3-MA abolished the protective effect of IPostC on mitochondria, many mitochondrial vacuoles appeared (as indicated by red arrows) (Figure 7A). In addition, mitochondrial membrane potential was measured to explore mitochondria function. The membrane potential, which was expressed by JC-1 polymer/monomer fluorescence ratio, was significantly lower in the H/R group than the control group, while it was reversed by HPostC. Autophagy inhibitor 3-MA abolished the effect of HPostC (Figure 7B). 

## 3. Discussion

I/R has a high morbidity and mortality in modern society. Therefore, there is an urgent need for effective clinical treatment to solve this problem. IPostC, defined as transient episodes of ischemia and reperfusion, applied at the onset of reperfusion, has been reported to effectively ameliorate I/R injury of the heart [23]. Autophagy is reported to be promoted during myocardial I/R and it may be involved in cardioprotection during ischemia. But it is not fully understood whether autophagy is regulated by IPostC and what role it plays. Several studies have shown that nNOS is involved in I/R and IPostC, and its expression may affect autophagy activity to a certain extent. However, the relationship among nNOS, autophagy and IPostC is not well understood. In the present study, we treated myocardial IPostC model recombinant with nNOS inhibitor L-VNIO or autophagy inhibitor 3-MA, and demonstrated that IPostC enhanced autophagic activity against reperfusion injury during early reperfusion, partially through the nNOS/AMPK/ mTOR signaling pathway both in vivo and in vitro.

Autophagy is a highly dynamic and multi-step process. Its activity is determined by autophagy flux. The implication of autophagy flux includes the whole process of autophagy, such as the formation of macrophages, the transport of substrates to lysosomes, the degradation of substrates and the release of macromolecules to the cytoplasm [24]. Therefore, it is necessary to monitor the autophagic flux to detect the activity of autophagy. Membrane-bound LC3-II is retained in mature autophagosomes and is not released until fusion with the lysosomal [25]. So, it can be used as a marker for the detection of autophagy. p62/SQSTM1, known as the key protein of protein degradation pathway, participates in the selective degradation of ubiquitinated protein by autophagy [26], so the expression of p62 directly reflects the level of autophagy clearance. Both the levels of LC3-II and p62 protein need to be detected in order to monitor the autophagic flux [27].

In this study, we detected the level of LC3-II and observed autophagosomes under electron microscopy. The formation of autophagosome increased in the I/R myocardium (Figure 1A,C), but a higher level of p62 indicated that the autophagy clearance was inhibited (Figure 1B), presented as an impaired autophagic flux due to impaired fusion with lysosomes or autolysosomal degradation. Therefore, autophagosomes increased during I/R perhaps owing to the reduced autophagosome clearance, and autophagic activity was not further enhanced during reperfusion phase, which was against our previous viewpoint. The LC3-II/LC3-I ratio was significantly greater and more autophagosomes were observed in the IPostC myocardium; these evidences indicate that IPostC induced autophagy and promoted the formation of autophagosomes. Meanwhile, the low level of p62 expression suggests that the autophagic flux was fluent and the progress of autophagy was completed; IPostC could promote autophagy clearance and enhance the autophagic activity. However, nNOS inhibitor L-VNIO abolished the effect of IPostC. The level of LC3-II was decreased and autophagosomes were rarely detected, meanwhile autophagy clearance was also inhibited. Therefore, the autophagic activity was suppressed in the IPostC + L-VNIO group. 

The above results were also validated in vitro. The autophagic flux was blocked during reoxygenation while it was restored after the application of HPostC. HPostC raised the autophagic activity but L-VNIO and nNOS-siRNA abolished the effect (Figure 3 and Figure S1). These data also suggest that IPostC enhanced autophagic activity through nNOS-mediated pathways in vitro. 

Under the condition of nutritional deficiencies, AMPK is activated due to an increase in the AMP/ATP ratio [28,29]. In the early stage of myocardial ischemia, the level of ATP decreases and AMPK is activated in cardiomyocytes, meanwhile autophagy is induced [28]. AMPK plays a vital role in the initiation of autophagy during myocardial ischemia phase. It acts as a nutritional sensor in regulating autophagy activity to deal with the energy crisis of myocardial ischemia [30]. The mTOR is negatively regulated by AMPK. The inhibition of mTOR will result in the induction of autophagy. Some studies have reported that autophagy is upregulated through activation of AMPK-mTOR signaling during myocardial ischemia. However, whether this signal pathway is involved in I/R and IPostC remains unclear. In this study, we found that I/R increased AMPK phosphorylation and reduced the level of p-mTOR. It suggests that I/R induced autophagy via the activation of AMPK and inhibition of mTOR despite a decrease in autophagy clearance during reperfusion (Figure 2B, Figure 3D and Figure 4). In the IPostC group, IPostC further increased the level of AMPK phosphorylation and reduced the level of p-mTOR, but L-VNIO abolished the effects (Figure 4A). These data suggest that activation of the nNOS-AMPK-mTOR signaling pathway was involved in the regulation of IPostC on autophagy during early reperfusion. According to our previous research and the results in this study, with the application of IPosC, the activaton of nNOS was restored and the generation of NO was increased. More NO generated can bind to and inhibit cytochrome synthase [31] and creatine kinase activity [32], thereby providing a mechanism to increase the AMP/ATP ratio and activate AMPK. Therefore, the level of p-AMPK was higher and autophagic activity was stronger in the IPostC group. These findings were also confirmed in H9c2 cells in vitro (Figure 4B). 

Autophagic activity was enhanced by IPostC during early reperfusion, but whether it played a beneficial role in the protection of IPostC is still up for discussion. 3-MA, a classical autophagy inhibitor, was used to investigate the contribution of autophagy to IPostC. In our study, IPostC significantly reduced the myocardial infarct size and protected the structure of myocardium and mitochondria compared with I/R, but 3-MA reversed these effects (Figure 5 and Figure 7A), suggesting that autophagy was involved in the cardioprotection of IPostC against I/R injury. Several studies have shown that decreased autophagosome clearance is detrimental to cardiomyocyte survival during reperfusion [11,33], thus we suppose that the recovery of the impaired autophagic flux is important in IPostC‑induced cardioprotection.

Under some pathological conditions such as I/R, a mass of generated ROS may cause oxidative stress and mitochondrial dysfunction. Oxidative stress could trigger autophagy and autophagy could reduce the level of oxidative stress to some extent. In our present study, excessive production of ROS rather than RNS at an early stage of reperfusion was reduced by HPostC, but the effect was suppressed by 3-MA (Figure 6A,B). This is probably because of activation of autophagy and unimpeded autophagy clearance by HPostC. In addition, HPostC could restore mitochondrial membrane potential to normal levels by enhancing autophagy activity to protect mitochondrial function and reduce the production of mitochondrially-generated ROS [34]. These data have indicated that autophagy played an important role in attenuating oxidative stress in IPostC.

To further confirm our hypothesis that promotion of autophagy activity being important for the cardioprotection of IPostC, we measured cell apoptosis and viability (Figure 6C–E). As a result, high cell apoptosis and reduced cell viability induced by H/R were restored by HPostC, while 3-MA reversed these effects. Therefore, autophagy was involved in the protection effect of IPostC against I/R injury. IPostC exerted its beneficial effect via promoting autophagy.

This study is the first to demonstrate that nNOS plays an important role in promoting autophagy in the cardioprotection of IPostC during early reperfusion. IPostC decreased oxidative stress and I/R injury partially by enhancing autophagy activity via the activation of nNOS/AMPK/ mTOR pathway during early reperfusion. Therefore, enhanced autophagy clearance and autophagy activity during early reperfusion maybe suitable for recovery from myocardial I/R injury.

## 4. Materials and Methods

### 4.1. Ethics Statement

All procedures were strictly performed in accordance with the regulations of the ethics committee of the International Association for the Study of Pain and the Guide for the Care and Use of Laboratory Animals (The Ministry of Science and Technology of China, 2006). All animal experiments were approved by the Nanjing Medical University Animal Care and Use Committee (Project identification code: SCXK-2011-0003, 6 December 2011), and were designed to minimize suffering and the number of animals used.

### 4.2. Reagents and Antibodies

Dulbecco’s modified Eagle’s medium (DMEM) and fetalbovine serum (FBS) were purchased from Gibco. LC3A/B, p62, anti-AMPK, p-AMPK (Thr172), anti-mTOR, p-mTOR antibodies were purchased from Cell Signaling Technology, Inc. (Danvers, MA, USA). Autophagy inhibitor 3-methyladenine (3-MA), GAPDH antibodies, Goat anti-rabbit and anti-mouse IgG-HRP were purchased from Sigma (St. Louis, MO, USA). The selective nNOS inhibitor L-VNIO was purchased from Enzo Life Sciences, PA, USA. p-nNOS^Ser852^ (Abcam, Cambridge, MA, USA), nNOS (Abcam, Cambridge, MA, USA), nitrotyrosine (Cell Signaling Technology) were used in Western blot. Mitochondria membrane potential assay kit with JC-1 (Beyotime Biotechnology, Nantong, China) was used to determine mitochondria membrane potential.

### 4.3. I/R Modeling

Myocardial I/R was induced by transient myocardial ischemia for 30 min and was followed by reperfusion for 1 h. Adult male C57/B6 mice (body weight around 25–30 g) were anesthetized with 4% chloral hydrate intraperitoneally (i.p.) (100 mg/kg). After a left lateral thoracotomy and pericardiectomy, the left anterior descending coronary artery was occluded for 30 min with an 8-0 nylon suture and two cotton coils were set under the suture to prevent arterial injury. IPostC was performed at the beginning of reperfusion, which was composed of 30 s of reperfusion and 30 s of ischemia for three cycles followed by 1 h reperfusion. Mice were pretreated with 3-MA (15 mg/kg; i.p.) 30 min before regional ischemia. The selective nNOS inhibitor L-VNIO (30 mg/kg; i.p.) was injected 15 min before reperfusion. Regional ischemia was confirmed by ST segment elevation on the electrocardiogram. 

### 4.4. Culture and Experimental Protocols for H9C2 Cells

H9c2 cells were cultured in high glucose Dulbecco’s modified Eagle’s medium supplemented with 10% fetal bovine serum. Cells were transferred to an airtight hypoxia container filled with 100% N_2_ maintained with 37 °C simulated hypoxia in vitro. Before the cells were moved to the hypoxia container, the culture medium was replaced with serum-free, glucose-free DMEM that had been saturated with N2 gas for 1 h. The general experimental protocols used are described below. Control group: H9c2 cells were cultured in 10% fetal bovine serum and high glucose DMEM in a normoxic incubator for the whole experiment process. H/R group: H9c2 cells were subjected to 1 h of hypoxia and 1 h of reoxygenation. During reoxygenation, cells were cultured with low-glucose DMEM containing 10% serum. Then cells were used for subsequent experiments. HPostC group: after hypoxia for 1 h, HPostC was carried out. HPostC contained three cycles of 5 min of hypoxia and 5 min of reoxygenation followed by reoxygenation. The medium was replaced by low-glucose DMEM containing 10% serum after hypoxia. In the hypoxic phase, the cells were transferred to the hypoxia container. After 5 min of hypoxia, cells were placed in an incubator for 5 min. After three cycles, cells were subjected to reoxygenation for 30 minutes. The selective nNOS inhibitor L-VNIO (10 μM) was administered at the beginning of reoxygenation. Incubation of 3-MA (5 mM) was 2 h before hypoxia.

### 4.5. Infarct Size Measurement

Hearts were removed and sliced from the top to the bottom of the heart. Then, each slice was incubated with 1.5% triphenyltetrazolium chloride (Sigma) at 37 °C for 20 min. The areas of infarct were analyzed using Image J software (National Institutes of Health, Bethesda, MD, USA).

### 4.6. Hematoxylin and Eosin Staining (HE)

The Mouse hearts were taken down and fixed in buffered 10% formalin for 24 h. Then they were embedded in paraffin, serially sectioned and stained with HE.

### 4.7. Intracellular ROS Detection

Reactive Oxygen Species Assay Kit (DCFH-DA, Beyotime Biotechnology) was used to detect the generation of ROS in H9c2 cells. After 1 h of hypoxia and 30 min of reoxygenation, the medium was discarded and washed with PBS. H9c2 cells were incubated with 10 μM DCFH-DA in PBS at 37 °C for 15 min. Then the cells were observed by a fluorescence microscope. Fluorescence intensity was analyzed by Image J software (National Institutes of Health, Bethesda, MD, USA).

### 4.8. Evaluation of Cell Death

Cell viability was assessed by CCK-8 kits. The medium volume of each hole in the 96 holes was 200 µL. 20 µL of CCK-8 solution was added to the culture medium before reoxygenation. Then the viability was measured by microplate reader at 450 nm. The apoptotic cells were measured by an Annexin V-FITC/PI Assay Kit (Sigma, St. Louis, MO, USA ). Firstly, the cells were washed with cold PBS and mixed in binding buffer, subsequently Annexin V-FITC and propidium iodide were added to the sample. Cells were incubated for 15 min in the dark at room temperature. Finally, the samples were analyzed using a FACSCalibur flow cytometer (BD Biosciences, San Jose, CA, USA). 

### 4.9. Western Blot

The left ventricular muscle and H9c2 cells in each group were lysed in RIPA lysis buffer (Beyotime Biotechnology) for 30 min on ice. Protein lysate were centrifuged at 12,000× *g* for 15 min. A Bicinchoninic Acid Protein Assay Kit (Beyotime Biotechnology) was used to measure the concentration of the lysates. Total protein (30 μg) was separated on 8%–15% SDS polyacrylamide gels and was transferred to polyvinylidene difluoride membranes. The membranes were incubated with protein antibodies overnight at 4 °C after blocking with 5% fat-free milk for 2 h at room temperature. Then the membranes were washed with TBST for 30 min and incubated with secondary antibodies for 2 h at room temperature. Immunoreactive bands were detected by enhanced chemiluminescence (Pierce, Rockford, IL, USA) and exposed by Kodak Image Station 4000 MM PRO (Carestream Health Inc., Rochester, NY, USA).

### 4.10. Transmission Electron Microscopy

Heart tissue pieces of 1 mm^3^ removed from the ischemic area at risk were fixed with 2.5% glutaraldehyde phosphate buffered saline, 1% osmium, and 0.1 mole/L tetroxide phosphate buffered saline, dehydrated with an acetone gradient, embedded in EPON-812 resin at 358C overnight, and polymerized at 608 °C for 48 h. Ultrathin sections (60–70 nm thick) were placed on grids (200 mesh), double stained with uranyl acetate and lead citrate, and viewed under a FEI (Tecnai G2 Spirit Bio TWIN) electron microscope (FEI, Portland, OR, USA).

### 4.11. GFP-LC3 Adenovirus Transfection Assay

For assessment of autophagosomes using epifluorescence imaging, cells plated on coverslips were transfected with GFP-LC3 adenovirus (Hanbio Biotechnology Co., Ltd., Shanghai, China) following the manufacturer’s instructions. Ten hours after transfection, the medium was changed. After 48 h, the cells were treated with hypoxia and reoxygenation. The selective nNOS inhibitor L-VNIO (10uM) was administered at the onset of reoxygenation for 1 h. Cells were subsequently fixed in 4% paraformaldehyde and mounted on slides with DAPI (Beyotime Biotechnology). Epifluorescence images were taken using Leica DM IRBE epifluorescence microscope (Leica Microsystems, Heidelberg, Germany) and images were processed using ImageJ software (National Institutes of Health, Bethesda, MD, USA).

### 4.12. Mitochondrial Membrane Potenital Detection

Preparation for JC1 working fluid according to the assay kit was completed before the experiment. The cells were collected and centrifuged at 1000× *g* for 10 min. After removing the supernatant, 0.5 mL of medium was added to the centrifuge tube. Then 0.5 mL of JC1 working fluid was added to mix with the cells. After the incubation of H9c2 cells in an incubator for 20 min, the cells were centrifuged at 600× *g* for 10 min. Afterwards, the cells were washed with JC-1 buffer (1×) twice. Finally, the samples were analyzed using a FACSCalibur flow cytometer (BD Biosciences, San Jose, CA, USA). The degree of depolarization of mitochondrial membrane potential was expressed as the ratio of red fluorescence to green fluorescence intensity.

### 4.13. Statistical Analysis

The data are expressed as the mean ± SEM. Experimental data were analyzed with Student’s *t*-test. *p* < 0.05 was deemed to be statistically significant. The software Prism GraphPad 5.0 (GraphPad Software Inc., San Diego, CA, USA) was applied to the data analysis.

## Supplementary Materials

Supplementary materials can be found at www.mdpi.com/1422-0067/18/3/614/s1.

## Abbreviations

I/R	ischemia/reperfusion
nNOS	neuronal nitric oxide synthase
IPostC	ischemic postconditioning
H/R	hypoxia/reoxygenation
HPostC	hypoxic postconditioning
AMPK	adenosine monophosphate-activated protein kinase
mTOR	mammalian target rapamycin
ROS	reactive oxygen species
IPre	ischemic preconditioning
SR	sarcoplasmic reticulum
NO	nitric oxide
HE	hematoxylin and eosin Staining
