# Supplementary material for: Myocardial Ischemic Postconditioning Promotes Autophagy against Ischemia Reperfusion Injury via the Activation of the nNOS/AMPK/mTOR Pathway
Source: Int J Mol Sci. 2017 Mar 11;18(3):614. doi: 10.3390/ijms18030614 (PMC5372630; doi:10.3390/ijms18030614)
Supplement: Supplementary file 1 [file ijms-18-00614-s001.pdf]

Supplementary Materials: Myocardial Ischemic Postconditioning Promotes Autophagy against Ischemia Reperfusion Injury via the Activation of the nNOS/AMPK/mTOR Pathway

Maojuan Hao, Suhua Zhu, Liang Hu, Hongyi Zhu, Xiaowei Wu and Qingping Li

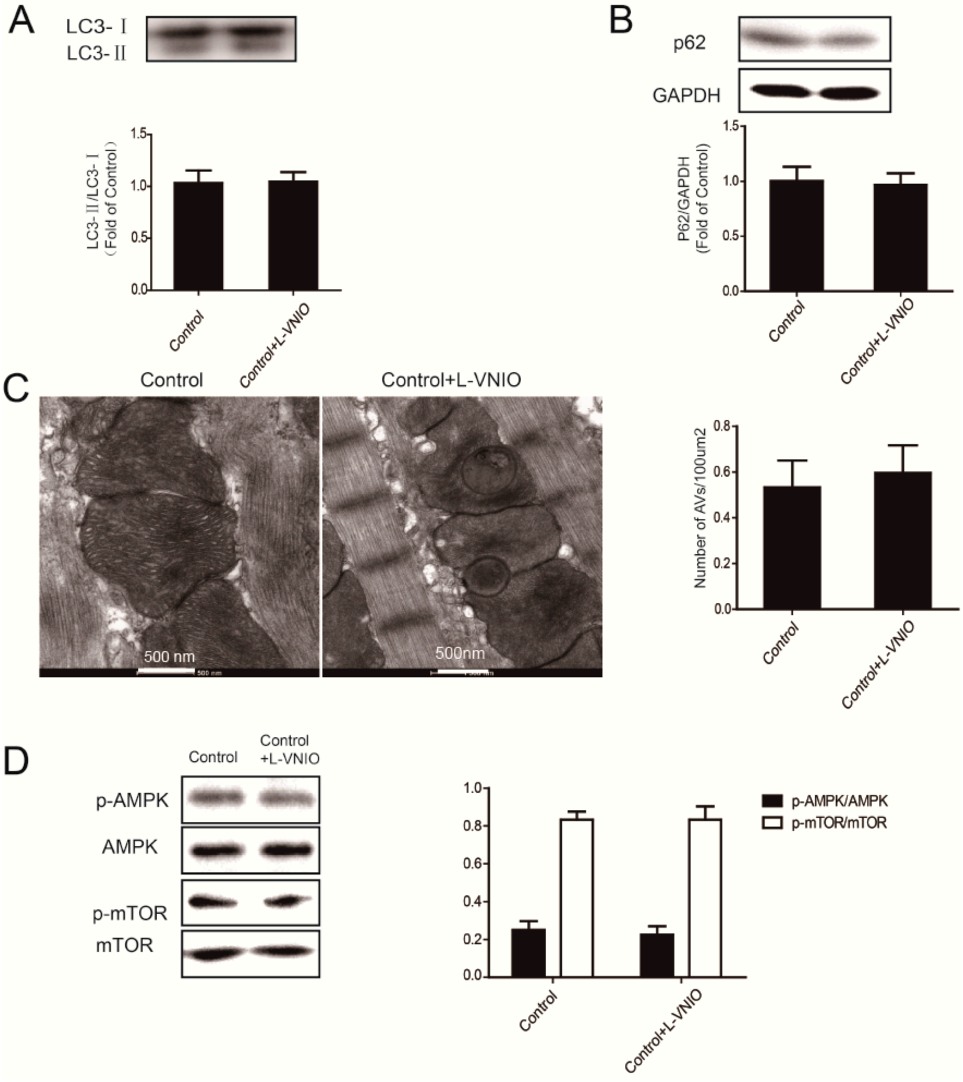

Figure S1. There was no significant difference in autophagy activity between the control group and the control +L-VNIO group in the myocardium. (**A**) The protein expression of LC3 in the myocardium at 30 min of reperfusion. (**B**) The level of p62 was detected in the myocardium. (**C**) Autophagosomes were observed using electron microscopy. (**D**) Expression of p-AMPK, p-mTOR in the myocardium at 30 min of reperfusion. The mean values ± SEM, $n = 6$.

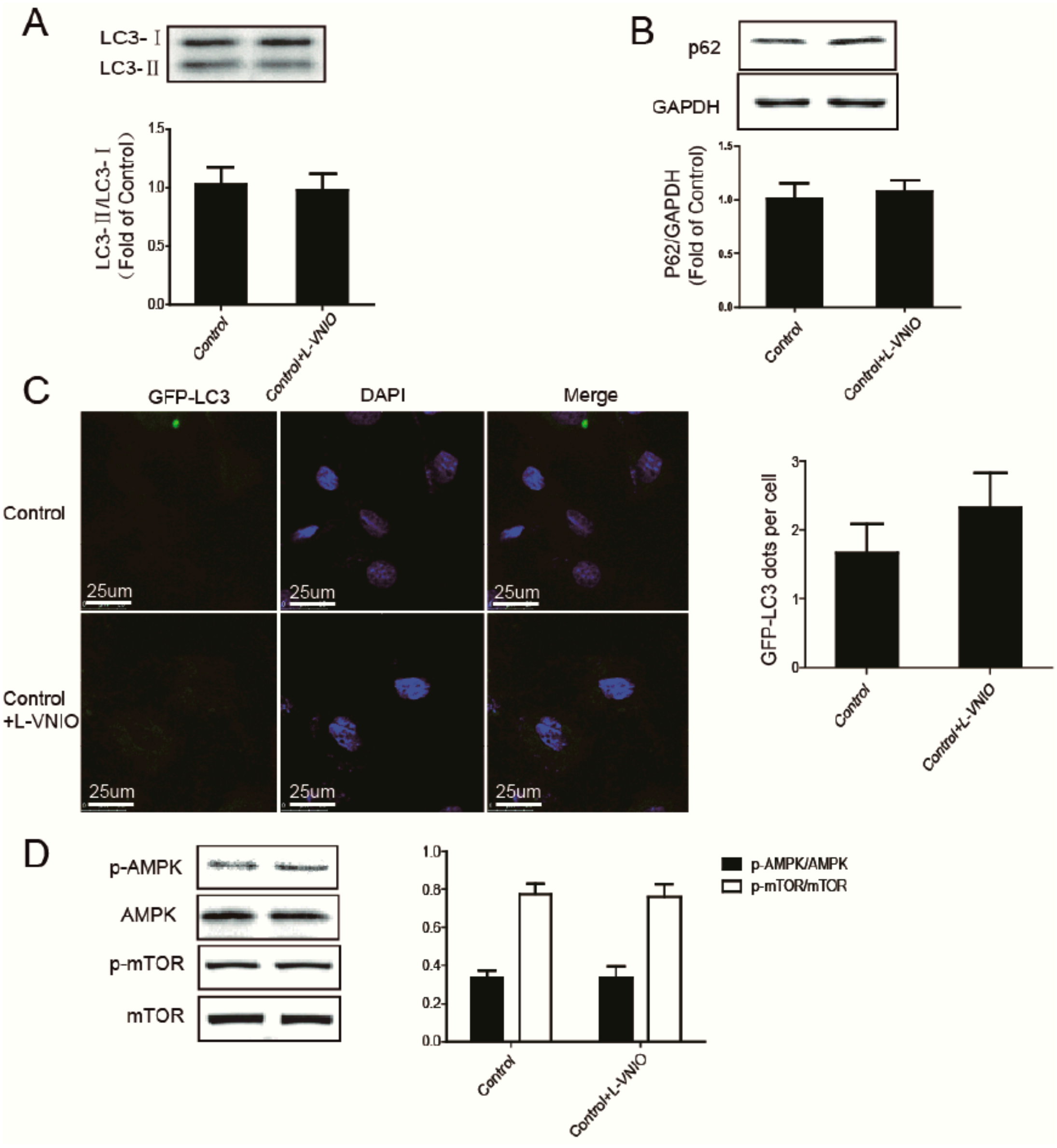

Figure S2. Autophagy activity was at a low level both in the control group and the control+L-VNIO group in H9c2 cells. (**A**) The protein expression of LC3 in cells at 30 min of reoxygenation. (**B**) Expression of p62 at 30 min of reoxygenation. (**C**) H9c2 cells were infected with GFP-LC3 adenovirus. The nuclei were labeled with DAPI (blue). (**D**) The protein expression and optical density analysis of p-AMPK (Thr172)/AMPK, p-mTOR (Ser2448) /mTOR. The mean values ± SEM, n = 6.

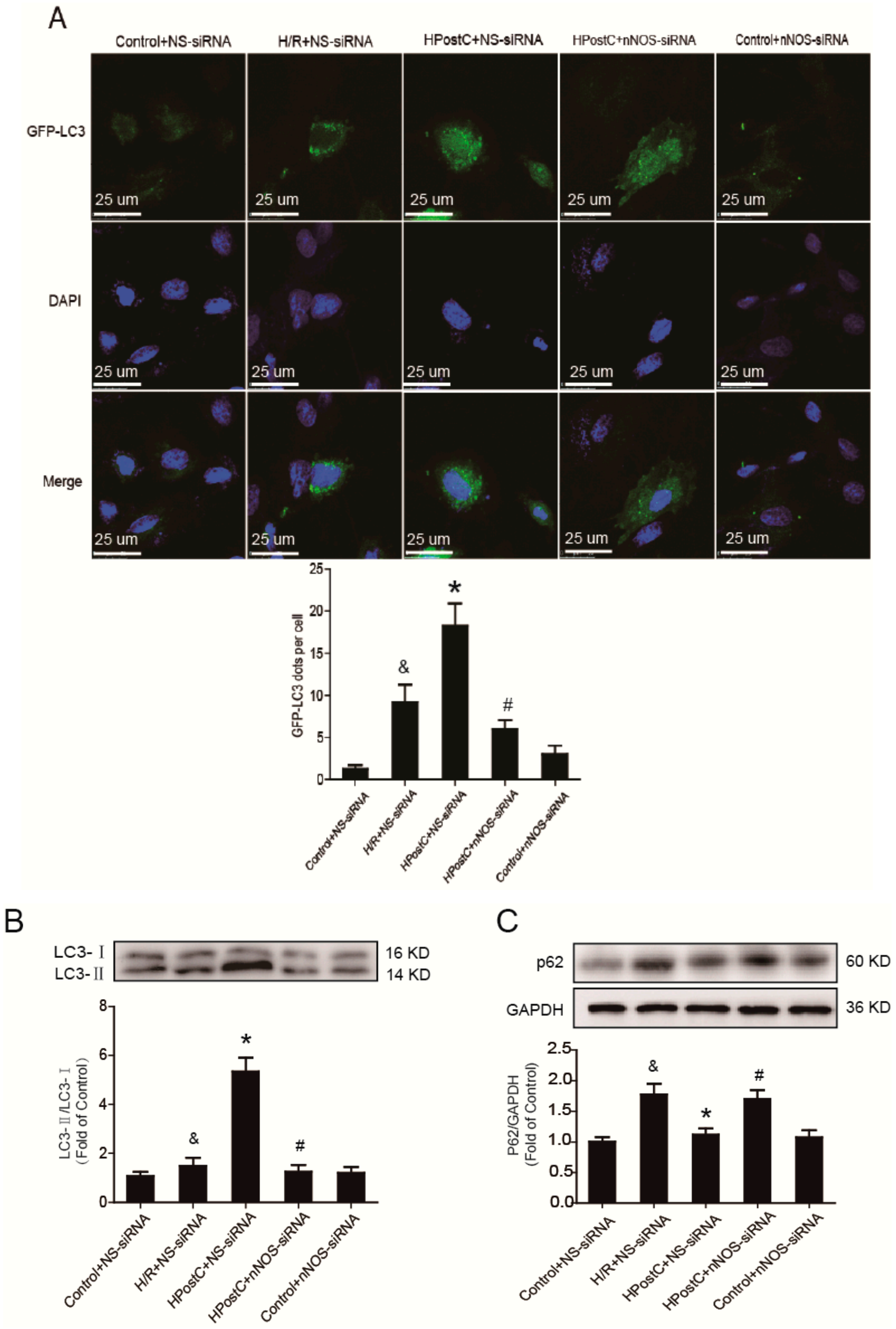

A
Control+NS-siRNA
H/R+NS-siRNA
HPostC+NS-siRNA
HPostC+nNOS-siRNA
Control+nNOS-siRNA
GFP-LC3
DAPI
Merge
25 um
GFP-LC3 dots per cell
B
LC3-Ⅰ
LC3-Ⅱ
16 KD
14 KD
LC3-Ⅱ/LC3-Ⅰ (Fold of Control)
C
p62
GAPDH
60 KD
36 KD
P62/GAPDH (Fold of Control)

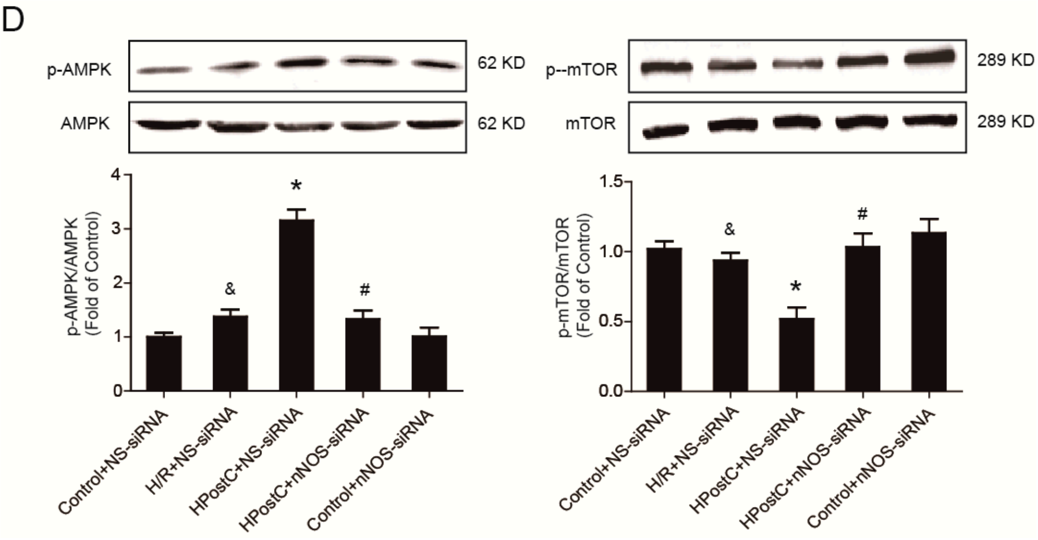

Figure S3. Hypoxic postconditioning (HPostC) enhanced autophagic activity via the activation of the nNOS/AMPK/mTOR pathway. (**A**) H9c2 cells were infected with GFP-LC3 adenovirus. The nuclei were labeled with DAPI (blue). (**B**) Expression of LC3 in H9c2 cells at 30 min of reoxygenation. (**C**) Expression of p62 at 30 min of reoxygenation. (**D**) Expression of p-AMPK, p-mTOR in H9c2 cells at 30 min of reoxygenation $^{\&}p < 0.05$ versus control, $^{*}p < 0.05$ versus hypoxia/reoxygenation (H/R), $^{\#}p < 0.05$ versus HPostC. The mean values ± SEM, $n = 6$.

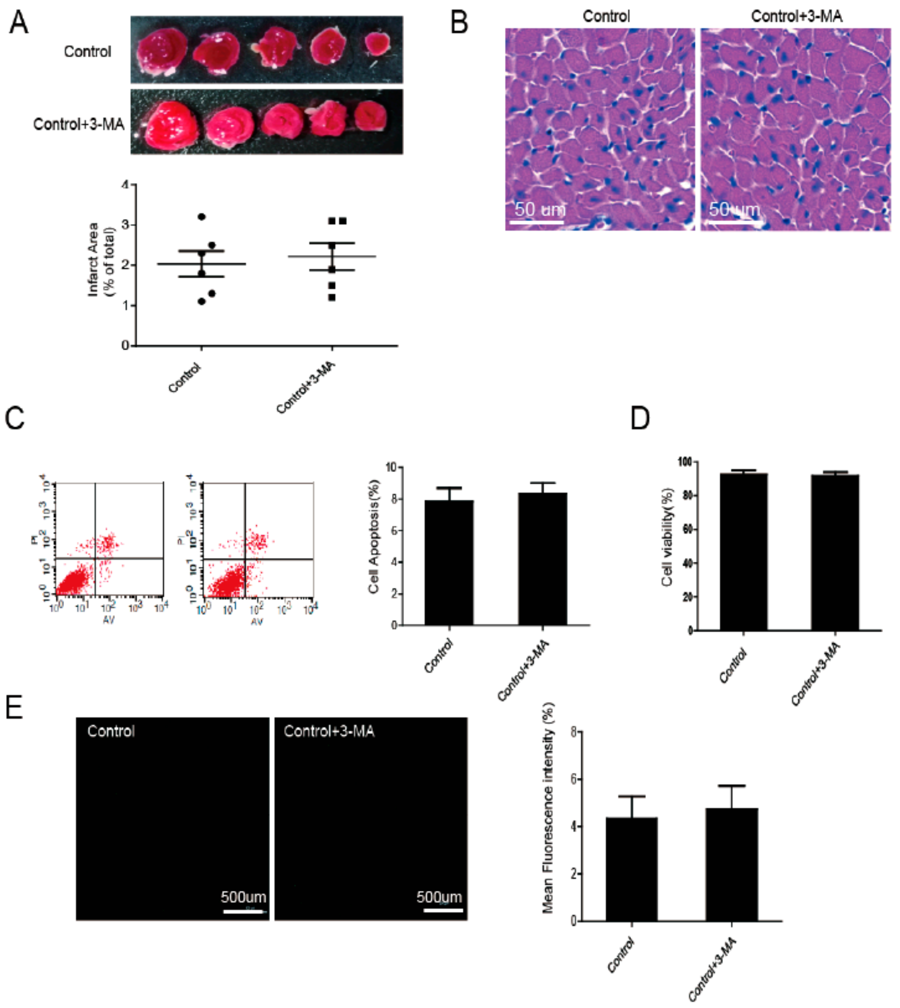

Figure S4. Autophagy inhibitor 3-MA has no effect on myocardium and H9c2 cells. (**A**) Infarct size was measured at 120 min of reperfusion. (**B**) Representative histological images of hearts at 120 min of reperfusion. (**C**) Determination of cell apoptosis. (**D**) Determination of cell viability. (**E**) ROS production was detected in H9c2 cells at 30 min of reoxygenation. The mean values ± SEM, *n* = 6.

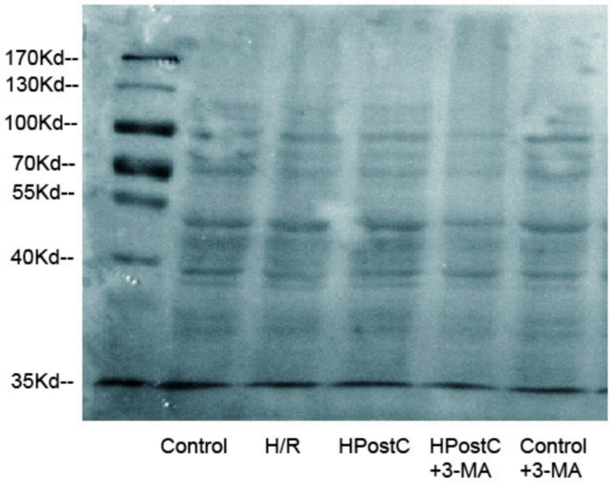

Figure S5. Assessment of nitrosative stress in H9c2 cells at 30 min of reoxygenation (n=6 per group).

Supplementary material and methods

Reagents and antibodies

Nitrotyrosine (Cell Signaling Technology) were used in Western blot.

Knockdown of nNOS using siRNA in H9C2 cells

The nNOS and negative control siRNAs were purchased from Shanghai GenePharma Co., Ltd. (Shanghai, China). The sequences of each siRNA were as follows: The sequences of each siRNA were as follows: nNOS (forward 5′-GCGAACAACUCCCUCAUUATT-3′ and reverse 5′-UAAUGAGGGAGUUGUUCGCTT-3), and the negative control (forward 5′-UUCUCCGAACGUGUCACGUTT-3′ and reverse 5′-ACGUGACACGUUCGGAGAATT-3′). Cells were plated at a density of 9 × 10^4 cells in small dishes. After siRNA was preincubated with Oligofectamine in serum-free Opti-MEM medium (Invitrogen Corporation) for 30 min, cells were transfected with nNOS or negative control siRNA for 5 h, then cells were incubated at 37 °C in incubator.
